# Effect of total parenteral nutrition on the duration of haemofilter circuit

**DOI:** 10.1186/cc9530

**Published:** 2011-03-11

**Authors:** S Saha, P Shah, J Gibbs, J Collins

**Affiliations:** 1 Broomfield Hospital, Chelmsford, UK

## Introduction

An effective haemofilter circuit is essential for performing continuous renal replacement therapy (CRRT) efficiently and without interruption. Premature clotting is a major problem in the daily practice of CRRT associated with blood loss [1], increased workload and cost implications. Early clotting is related to various factors ranging from bio-incompatibility of the CRRT circuit material, the modality used, ineffective anticoagulation, to site of catheter placement. Shortened haemofilter circuit survival time due to high lipid content in total parenteral nutrition (TPN) has also been described in a case report [2]. We wish to determine whether TPN infusion led to shortening of haemofilter circuit duration.

## Methods

We conducted a retrospective analysis of notes of patients who had undergone CRRT in an adult general ICU over 2 years. Demographic (age, sex) and clinical (platelet count, INR, APTT, anticoagulant used and the rate of infusion of anticoagulant) data that are known to influence the duration of CRRT circuit were compared. Cycles terminated because of high Pin pressure or documented failure of the circuit were included in the study and the duration of the circuit was determined. Note was made if the patient was on TPN during CRRT. They were divided in two groups: CRRT with TPN, and CRRT without TPN. All patients had the similar make vascath (14Fr, polyurethane catheter; Logitech) and the same CRRT machine and circuit.

## Results

One hundred and twenty-one patients had undergone CRRT in the unit in the past 2 years. In total, 246 CRRT circuits were used. A linear regression model was fitted to the duration of filtration with TPN as a categorical predictor, along with other covariates. The mean duration of haemofilter circuit was 24.51 (24.08 to 29.08) hours without TPN and 17.22 (14.98 to 23.59) hours on TPN. With the maximal model, TPN use was significantly (*P *< 0.002) associated with a decrease in duration of filtration, but none of the other factors were significant. There was a tendency for platelet count to be significant.

## Conclusions

So considering the effect sizes, both TPN and increase in platelet count were associated with significant reduction in the duration of haemofiltration circuit. TPN led to decrease in duration of the haemofilter circuit by 7 hours. The effect of TPN was found to be independent of the platelet count.
